# High risk of developing dementia in Parkinson’s disease: a Swedish registry-based study

**DOI:** 10.1038/s41598-022-21093-8

**Published:** 2022-10-06

**Authors:** Daniel Oudin Åström, Jacob Simonsen, Lars Lau Raket, Simona Sgarbi, Johan Hellsten, Peter Hagell, Jenny M. Norlin, Klas Kellerborg, Pablo Martinez-Martin, Per Odin

**Affiliations:** 1 H Lundbeck A/S, Ottiliavej 9, 2500 Valby, Denmark; 2 Clinical Memory Research Unit, Department of Clinical Sciences, Lund University, Lund, Sweden; 3 The PRO-CARE Group, Faculty of Health Sciences, Kristianstad University, Kristianstad, Sweden; 4 The Swedish Institute for Health Economics, Lund, Sweden; 5 Center for Networked Biomedical Research in Neurodegenerative Diseases (CIBERNED), Carlos III Institute of Health, Madrid, Spain; 6 Division of Neurology, Department of Clinical Sciences, Lund University, Lund, Sweden

**Keywords:** Dementia, Neurology, Neurological disorders, Movement disorders, Parkinson's disease

## Abstract

Dementia have substantial negative impact on the affected individual, their care partners and society. Persons living with Parkinson’s disease (PwP) are also to a large extent living with dementia. The aim of this study is to estimate time to dementia in PD using data from a large quality register with access to baseline clinical and patient reported data merged with Swedish national health registries. Persons with Parkinson’s disease in the Swedish Neuro Registries/Parkinson’s Disease Swedish PD Registry (PARKreg) in Sweden were included and linked to national health registries and matched by sex and age to controls without PD. Time to dementia was analysed with Cox regression models assuming proportional hazards, with time since diagnosis as the underlying time variable. In this large prospective cohort study, PwP had approximately four times higher risk of developing dementia as compared to age and sex-matched controls, a finding which remained after adjusting for potential confounders. The present results underline the high risk of dementia in PD and further emphasize the importance of developing symptomatic and ultimately disease modifying strategies to counteract this part of the non-motor symptomatology in PD.

## Introduction

Dementia causes a substantial negative impact on the affected individual, their care partners and society. The affected individual will lose their ability to function independently and face a reduced survival and health related quality of life, care partners experience diverse degrees of burden and increasing distress^1–3^ as well as reduced quality of life, and dementia impacts society at large, with high societal costs and burden on the health care sector^4,5^. According to several population based epidemiological studies 20 to 40% of individuals living with Parkinson’s disease (PD) also have dementia^6–11^. Incidence rates are increased 4–6 times as compared to non-PD controls, and the cumulative prevalence is very high. Recently, a Swedish study reported the 10 year cumulative incidence to be approximately 54%^12^. Other studies have reported that at least 75% of people with PD (PwP) who survive for more than 10 years will develop dementia^9,13^.

Parkinson’s disease dementia (PDD) is considered a type of Lewy body dementia, but the course of disease and symptom manifestation is distinct from dementia with Lewy bodies (DLB)^14^. PDD is characterized by impaired executive and visuo-spatial functions as well as cognitive and memory impairment^15^. Beyond age, sex, daily levodopa dose, and sleepiness^16–18^, studies have suggested hallucinations^19^, apathy^20^, orthostatic hypertension^21^, visuospatial and visuo-perceptual errors^22^ to be predictive of PDD.

Furthermore, cognitive decline and dementia in PD may also arise due to Alzheimer’s disease co-pathology. This is evidenced by the finding that lower CSF Aβ42 concentrations has consistently been found to be associated with worse cognitive function and being predictive of future dementia in PwP^23^. However, in PDD in general, biomarkers of Alzheimer’s disease have not been consistently found to be abnormal^23–25^. Several large randomized controlled trials on the treatment of cognitive impairment or dementia in Parkinson´s disease and dementia with Lewy bodies have been performed. While it has been found that cholinesterase inhibitors and memantine improve global impression, and cholinesterase inhibitors enhance cognitive function, the benefit is limited and of a symptomatic nature^26^.

Swedish Neuro Registries/Parkinson’s Disease Swedish PD Registry (PARKreg), continuously follow-up PwP in clinical practice (see https://www.neuroreg.se) and contains detailed information on demographics, diagnosis, treatments, as well as clinical outcome assessments. The detailed level of information provided by the quality register, national registers as well as the size of the cohort in comparison to previous studies will help advance understanding of dementia in PD. Therefore, the aim of this study is to estimate time to dementia in PD using data from a large quality register with access to baseline clinical and patient reported data merged with Swedish national health registries.

## Materials and methods

### Sample

Swedish Neuro Registries/Parkinson’s Disease Swedish PD Registry (PARKreg), established in 2011, have continuous follow-up of clinical and patient-reported outcomes in clinical practice and through research (see https://www.neuroreg.se). The register currently covers approximately 9650 PwP, out of whom 6970 are still alive and residing in Sweden (of an estimated total of 22,000 PwP) and includes demographic variables, diagnosis, treatments, and physician reported clinical assessments of disease severity, and patient reported outcomes. The information included reflect clinical practise and generally, visits are on a yearly basis, however more frequent data entries is possible.

Two study cohorts were defined: persons with a diagnosis of PD (PD cohort) and a random sample of the general population without a diagnosis of PD (controls).

Data on 1581 PwP residing in the southernmost region of Sweden, Scania, who in addition had agreed to be included in PARKreg was accessed in April 2020. The Scania cohort of PARKreg covers approximately 50% of persons diagnosed with PD in the region. The PD cohort had the index date defined as the date of PD diagnosis recorded in PARKreg.

The control cohort consisted of a random selection from the general Scania population, matched by age and sex on a 1:5 ratio. For controls, the index date was defined as the date of PD diagnosis for their matched individual in the PD cohort.

Data on date of visit as well as the primary and secondary reason for visits to an in- or out-patient setting as well as visits to the primary health care were collected and was linked to the PD and control cohorts by the unique personal identification number (PIN). Data on date of death were linked to the PD and control cohorts by PIN from the Swedish cause of death registry.

Dementia was defined as a health care visit (in and/or outpatient visit or visit to primary health care) where any of the following ICD-10 codes were recorded, F00–F03.

F00: Alzheimer’s Dementia

F01: Vascular Dementia

F02: Dementia in other diseases classified elsewhere (includes PDD)

F03: Unspecified Dementia.

In a first sensitivity analysis we required persons to have at least two dementia coded health care visit to ascertain “real” dementia cases.

In a second, stand alone, sensitivity analysis we defined dementia as being prescribed an anti-dementia drug (ATC class N06D). Data of prescribed drugs were collected from the prescribed drug register and linked to the PD and control cohorts by the PIN.

As a third sensitivity analysis we combined the definition based on ICD-10 codes with the medication-based definition.

### Outcome variable

Time to dementia was defined as the time from PD diagnosis to the first health care visit with a dementia coded event. In the second sensitivity analysis, time to dementia was defined as time from PD diagnosis to the first prescription of an anti-dementia drug.

Follow-up time was defined as either time to dementia, loss to follow-up, time to death or end of study period (31 December 2019), whichever came first. Prevalent dementia cases were not included in the analysis.

### Statistical methods

Time to dementia was analysed with Kaplan–Meier plots as well as with Cox regression assuming proportional hazards, with time since diagnosis as the underlying time variable, resulting in Hazard Ratios (HR) and their 95% Confidence Intervals (CI). We also investigated age at onset as the underlying time variable (results almost identical).

Tests for proportionality were carried out by formal testing of the assumption of proportional hazards with an interaction term between the logarithm of time and the group variable.

We performed crude analyses and in additionally adjusted models we controlled for sex, year of birth, family socio-economic status (above/below median family income, collected from the Longitudinal integrated database for health insurance and labour market studies (LISA)) and marital status (collected from Statistics Sweden).

To take full advantage of the unique combination of clinal assessments and register data we stratified the analyses by baseline severity of disease as assessed by the Hoehn and Yahr scale. Mild PD was defined as PwP staged between 0.0 and 2.0, whereas moderate to severe PD was defined as PwP staged above 2.0^27^. We also investigated whether baseline severity modified the association. We calculated this as the ratio between the HR for the severe PD group at baseline and the HR from the reference group, i.e. mild PD group at baseline^28^.

As this is an observational study, unmeasured confounding may be present. We therefore calculated the E-value for the HRs and their lower bound. The E-value is a measure of the strength an unmeasured confounder would have to have with both the exposure and outcome to explain away the observed associations^29^.

SAS version 9.4 and R version 4.1.1 were used. Package Survminer was used to generate Kaplan–Meier survival plots^30^.

The study was performed in accordance with the Declaration of Helsinki and was approved by the Swedish Ethical Review Authority, Lund, Sweden (Dnr 2013/374 and 2019/05791). All individuals have given their informed consent to participate in PARKreg.

## Results

Data on 1581 persons with PD in PARKreg were retrieved in April 2020. Table 1 presents the sample characteristics at baseline. 1442 PwP had a recorded date of diagnosis of PD and 1369 PwP were dementia free at the date of diagnosis and were included in the time to event analyses.

**Table 1:** Baseline characteristics of the study participants.

	Persons with Parkinson’s disease			
	N total		N total	
Male gender, n (%)	1581	981 (62%)	7905	4900 (62%)
Birth year, median [IQR]	1581	1944 [1938, 1949]	7905	1944 [1938, 1949]
Age at baseline, median [IQR]	1581	72 [66, 77]	7905	72 [66, 77]
Age at onset, median [IQR]	1311	65 [57, 71]		
Age at diagnosis, median [IQR]	1442	67 [59, 73]		
Years since onset, median [IQR]	1311	6 [3, 10]		
Years since diagnosis, median [IQR]	1442	4.2 [1.6, 8.6]		
**Hoehn and Yahr stage at baseline, median [IQR]**	1305	[1.5, 3.0]		
Hoehn and Yahr ≤ 2.0, median [IQR]	751	[1.0, 2.0]		
Hoehn and Yahr > 2.0, median [IQR]	554	3.0 [2.5, 3.0]		
**Follow up time PD cohort (years), mean [SD]**	1362	9.4 [6.1]	6846	[6.3]
Hoehn and Yahr ≤ 2.0, mean [SD]	695	[4.8]		
Hoehn and Yahr > 2.0, mean [SD]	504	11.6 [6.8]		
**Marital status**	1559		7784	
Married		1023 (66%)		4518 (58%)
Single/unmarried		362 (23%)		2291 (29%)
Widowed		172 (19%)		971 (12%)
Family disposable income (1000 SEK), median [IQR]	1599	334 [218, 517]	7784	291 [178, 469]

*IQR* interquartile range, *SD* standard deviation.

During the study period 683 individuals were diagnosed with dementia, out of which 642 individuals had more than one record of dementia in the registries. Approximately 47% and 29% of individuals had their first diagnose of dementia in a primary health care setting and an in/out-patient setting respectively. Of those diagnosed in the primary health care setting, approximately 37% hade their first encounter in a memory clinic. Changing the definition of dementia to having been prescribed a N06D anti-dementia drug resulted in 780 individuals with dementia, out of which 160 were not, at the end of the study, diagnosed with dementia.

Table 2 presents the frequencies by category, group, and dementia definition.

**Table 2:** Frequency of dementia, by dementia category.

	1 or more records (main analysis)			2 or more records (sensitivity analysis 1)			N06D prescriptions (sensitivity analysis 2)		
	PD	Controls	All	PD	Controls	All	PD	Controls	All
Alzheimer’s dementia	5	65	70	5	64	69	6	53	59
Dementia elsewhere	185	13	198	178	13	191	184	10	194
Other dementia	65	225	298	55	207	262	64	210	274
Vascular dementia	10	83	104	10	77	87	10	83	93
Medication but no dementia							123	37	160
Total	265	386	651	264	248	609	387	393	780

**Figure 1:**
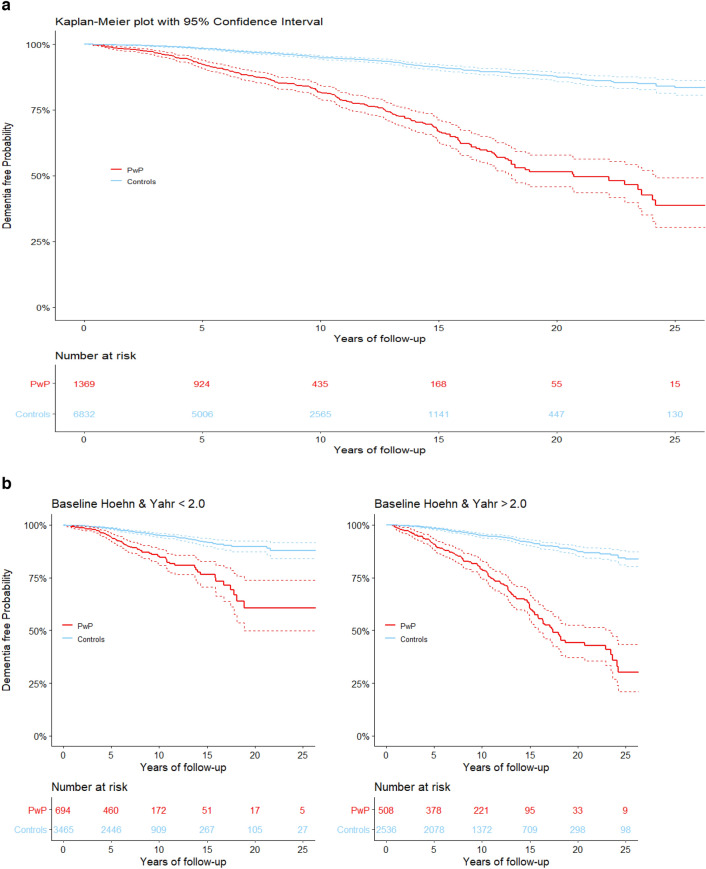
Kaplan–Meier plot of the probability of being dementia free.

Figure 1a,b presents the Kaplan–Meier plot of time to dementia in PwP vs. their matched controls for the main analyses for the total population and stratified on baseline Hoehn & Yahr staging restricted to 25 years after diagnosis, respectively.

Table 3 presents the HRs for PwP compared to their matched controls and their 95% Confidence Intervals (CIs) for the Cox proportional hazard models for the main and sensitivity analyses. The interaction term between the logarithm of time and the group variable was non-significant at the 5% level indicating that the proportionality assumption may be valid for the main models as well as for the sensitivity analyses.

**Table 3:** Hazard ratios and 95% confidence intervals for time to dementia.

	Main analysis		E-value	Sens. analysis 1	E-value	Sens. analysis 2	E-value
		HR (95% CI)	Lower limit	HR (95% CI)	Lower limit	HR (95% CI)	Lower limit
Full population	Crude	3.6 (3.1–4.2)	6.7 (5.7)	3.5 (3.0–4.2)	6.5 (5.5)	4.7 (4.0–5.5)	8.9 (7.5)
	Adjusted	3.7 (3.2–4.3)	7.1 (5.9)	3.7 (3.1–4.3)	7.1 (5.7)	4.8 (4.1–5.7)	9.1 (7.7)
Hoehn & Yahr ≤ 2.0	Crude	3.1 (2.4–4.1)	5.1 (4.2)	3.0 (2.3–3.9)	5.5 (4.0)	3.6 (2.7–4.7)	6.7 (4.8)
	Adjusted	3.4 (2.6–4.5)	6.3 (4.6)	3.1 (2.3–4.1)	5.7 (4.0)	3.7 (2.8–5.0)	6.9 (5.0)
Hoehn & Yahr > 2.0	Crude	5.1 (4.1–6.4)	9.7 (7.7)	4.3 (3.5–5.4)	8.1 (6.5)	6.2 (5.0–7.7)	11.9 (9.5)
	Adjusted	5.4 (4.3–6.8)	10.3 (8.1)	4.4 (3.5–5.6)	8.3 (6.5)	6.1 (4.9–7.7)	11.7 (9.3)

*HR* hazard ratio, *CI* confidence interval, *Sens. analysis 1* 2 or more records of a dementia coded event, *Sens. analysis 2* dementia defined prescribed a N06D anti-dementia drug.

The HRs from the third sensitivity analyses were consistently in the range between the HRs generated in the main analyses and the HRs generated in the second sensitivity analyses (results not shown).

There was suggestive evidence of increased risks of dementia based on Hoehn & Yahr staging at time of registration in PARKreg vs controls. The ratios of HRs (95% CI) for the main analysis for Hoehn & Yahr staging above 2.0 vs. 2.0 or below were 1.7 (1.2–2.3) and 1.6 (1.1–2.3) for the crude and adjusted models, respectively. The same ratios were for sensitivity analysis 1, 1.4 (1.0–2.1) and 1.4 (1.0–2.1) and for sensitivity analysis 2 they were, 1.7 (1.2–2.5) and 1.6 (1.4–2.3), which supports the results from the main analysis.

Supplementary table 1 presents all the model estimates along with their 95% CIs in the full population for the adjusted main and sensitivity analyses.

## Discussion

In this large prospective cohort study PwP had approximately 3.5–6.1 times higher risk of developing dementia as compared to age and sex-matched controls, depending on definition, a finding which remained after adjusting for potential confounders. Adding a restriction on having two or more diagnoses of dementia in the registries yielded similar results indicating that our results are robust, and we are ascertaining true dementia cases. Our results are in line with previous findings, for instance, as reviewed by Aarsland and Kurz (2009), 4–6 times higher incidence rates of dementia in PwP as compared to controls were reported^9^. The size of the PwP cohort used in the present study is, to the best of our knowledge, the largest to date, thus providing additional robustness to previous findings mentioned above.

Stratifying the analyses by Hoehn & Yahr staging at the first visit resulted in statistically significantly higher estimates for those with a Hoehn & Yahr staging above 2 as compared to those with a staging of 2 or below. We found that those with Hoehn & Yahr staging above 2, on average, had a longer time since being diagnosed with PD to being included in the register, thus our findings likely reflect that this group have further progressed disease pathology, which is related to increased risk of developing dementia. This group also had more time to be diagnosed with dementia during follow-up. In addition, they were older, an additional risk factor for developing dementia^10^. In all models, year of birth was significant with a negative coefficient, suggesting increased risk of being diagnosed with dementia with age.

When we changed the definition of dementia to having been prescribed an anti-dementia drug (ATC class N06D), we observed an increase in the number of dementia cases, as well as higher HRs. One possible explanation for the increased number of presumed dementia cases is that anti-dementia drugs (cholinesterase inhibitors) such as rivastigmine have been trialled with trending positive effects on gait stability and fall frequency in PwP, and may therefore have been prescribed off-label for those purposes^31^. Another indication for starting treatment with rivastigmine among PwP might be hallucinations, as reviewed by Samudra et al^32^. Using this definition, we may be misclassifying some non-dementia cases as dementia cases, illustrated by the fact that 160 individuals were prescribed anti-dementia drugs despite not having a dementia diagnose during the study. On the other hand, being prescribed an anti-dementia drug may increase functioning and ultimately delay diagnosis of dementia.

Recent work exploring the impact of age and time since PD onset on symptom profiles in PARKreg found interaction effects of aging and PD progression such that persons with higher age at onset experienced more severe symptom profiles faster^33^. For example, cognitive problems, as assessed by the cognitive status component of the CISI-PD scale, increased slightly with age, but a clear age-by-time since onset interaction was present suggesting that persons with later onset on average experienced cognitive problems faster as their disease progressed. Furthermore, presence of memory problems, as assessed by the forgetting recent events symptoms item of the Non-Motor Symptom Questionnaire, had a nonlinear trajectory with age, but also a clear age-by-time since onset interaction where PwP with later onset on average experienced memory problems sooner after onset. This is in line with the notion that dementias at large are age-related syndromes but may also point to a role for interactions between alpha-synuclein pathology with other age-related co-pathologies to cause PDD^34^.

Recent research using the same cohort of PwP showed that advanced and late stages of PD were associated with significant societal costs and that a large part of the costs were associated with formal care, such as nursing home and home care, as well as informal care provided by caregivers^35^. Hoskins et al. recently reported cognitive decline to be a factor associated with nursing home placement, which is a major cost-driving factor^36^. The extent to which costs related to dementia and cognitive decline in PwP are associated with dementia should be the focus of future research.

The cause of cognitive decline in PD is still debated, but that it is likely that the direct cortical involvement of Lewy body pathology is a major factor^37^. In addition, the degree of AD pathology in PD dementia cases are variable. The roles of cerebral amyloidosis in synucleinopathy-related cognitive impairments is still debated^38^. Further studies on these factors are needed, which remains utterly important when developing new treatment strategies.

### Strengths and limitations

The current study has several strengths. A major strength is the size of the cohort of PD patients as well as the ability to match the PwP to controls at the time of inclusion. In addition, we used the unique personal identification number to link the quality register where clinical scales of disease severity in PD is recorded to national registers enabling us to find incident dementia cases as well as calculate a precise follow-up time. In the current study we had access to, not only the nationwide in and outpatient register, but also to primary health care data, an additional advantage when it comes to the granularity of both diagnoses of PD as well as follow-up time. The linkage also allowed us to derive different estimates of the risk of developing dementia depending on progression of disease. The positive predictive value (PPV) in the Swedish inpatient registry has been reported in the range of 85–95%, however with differences between diagnoses in the inpatient registry^39^. For dementia, Jin et al. 2004 reported a PPV of 72%^40^.

As this is a retrospective observational register-based study, limitations must be acknowledged. The individuals diagnosed with PD that are part of PARKreg are seen on a regular (often yearly) basis by a trained neurologist, thus there may be a risk of confounding by indication on the diagnosis of dementia. There may be several reasons why PwP would receive a dementia diagnosis earlier than normal controls which are unrelated to the biology of PD but because of healthcare visits, caregivers' behavior, support programs etc. For instance, PwP in PARKreg are already being closely followed in the healthcare system. For individuals not already "in the system" a diagnosis of dementia may be delayed. In addition, unmeasured confounding may be present. To evaluate the impact, we quantified the strength of association an unmeasured confounder would have to have with both the exposure and the outcome to explain away the observed HRs. For the full cohort, the E-value for the adjusted model was 7.1, suggesting that an unmeasured confounder (besides those already adjusted for) would need to be associated with both PD as well as dementia of a similar magnitude, in order to explain away the observed HR in the study. Another potential limitation is that PwP may not have been assessed by the same neurologist at all visits. An additional limitation might be that we only included PwP from the southernmost region of Sweden. The prevalence of PD is very similar in Scania as in different parts of Sweden. Furthermore, the demographics of the study area should be representative for Sweden as age, sex and urban/rural distributions are similar, however with a larger proportion of the population born or having parents born outside of Sweden. However, the availability of neurologists varies in the country, with Scania having one of the higher numbers. This may result in dementia remaining undetected for a longer period of time in other parts of the country with less neurology capacity. Finally, the coverage of PARKReg of around 50% of the PwP in Scania in the current study poses another limitation as it unknown if the PwP in the registry have the same disease characteristics as compared to the total population.

## Conclusion

The present results underline the high risk of dementia in PD and further emphasise the importance of developing symptomatic and ultimately disease modifying strategies to counteract this part of the non-motor symptomatology in PD.

## Supplementary Information

Supplementary Table 1.

## Data Availability

The data that support the findings of this study are available from the corresponding author upon reasonable request.
