# Supplementary material for: High risk of developing dementia in Parkinson’s disease: a Swedish registry-based study
Source: Sci Rep. 2022 Oct 6;12:16759. doi: 10.1038/s41598-022-21093-8 (PMC9537530; doi:10.1038/s41598-022-21093-8)
Supplement: Supplementary file 1 — Supplementary Table 1. [file 41598_2022_21093_MOESM1_ESM.docx]

Supplementary Table 1. Parameter estimates

| **Supplementary Table 1** | | | | | | |
| --- | --- | --- | --- | --- | --- | --- |
|  | **Main analysis** | | **Sensitivity analysis 1** | | **Sensitivity analysis 2** | |
| Parameter | **Estimate** | **S.E** | **Estimate** | **S.E** | **Estimate** | **S.E** |
| PD vs control | 1.307 | 0.082 | 1.303 | 0.085 | 1.575 | 0.084 |
| Sex (ref = Female) | 0.219 | 0.087 | 0.212 | 0.090 | 0.221 | 0.089 |
| SES (ref = Low) | -0.076 | 0.097 | -0.028 | 0.101 | -0.010 | 0.100 |
| Year of birth | -0.096 | 0.006 | -0.099 | 0.006 | -0.098 | 0.006 |
| Civil status not included due to many levels  S.E: Standard Error | | | | | | |
